# Deciphering Silence: Functional Studies of GCK Synonymous and Nonsense Variants and Their Importance in Understanding Diabetes

**DOI:** 10.3390/genes17020214

**Published:** 2026-02-10

**Authors:** Concetta Aloi, Alessandro Salina, Serena Cappato, Nicola Minuto, Giuseppe D’Annunzio, Fabio Gotta, Davide Maggi, Paola Mandich, Laura Musso, Renata Bocciardi

**Affiliations:** 1 LABSIEM (Laboratory for the Study of Inborn Errors of Metabolism), Pediatric Clinic and Endocrinology Unit, IRCCS Istituto Giannina Gaslini, 16147 Genoa, Italy; concettaaloi@gaslini.org (C.A.); alessandrosalina@gaslini.org (A.S.); 2 Medical Genetics Unit, IRCCS Istituto Giannina Gaslini, 16147 Genoa, Italyrenata.bocciardi@unige.it (R.B.); 3 Regional Center for Pediatric Diabetes, IRCCS Istituto Giannina Gaslini, 16147 Genoa, Italy; 4 Medical Genetics Unit, IRCCS Ospedale Policlinico San Martino, 16132 Genoa, Italy; 5 Endocrinology Unit, Department of Internal Medicine and Medical Specialties, School of Medical and Pharmaceutical Sciences, University of Genoa, 16132 Genoa, Italy; davide.maggi@unige.it; 6 Department of Neuroscience, Rehabilitation, Ophthalmology, Genetics, Maternal and Child Health (DINOGMI), University of Genoa, 16132 Genoa, Italy; 7 Endocrinology Unit, IRCCS Ospedale Policlinico San Martino, 16132 Genoa, Italy

**Keywords:** MODY2, GCK, synonymous variant, splicing, precision medicine, minigene

## Abstract

**Background:** The most common form of monogenic diabetes is maturity onset diabetes of the young (MODY). This study investigates the molecular basis of MODY type 2 (*GCK*-MODY) in a group of Italian patients, focusing on the functional characterization of a synonymous variant, c.579G>T (p.Gly193Gly), in the glucokinase gene (*GCK*). **Methods:** Clinical evaluation and genetic analysis, including whole exome sequencing and Sanger sequencing, were used to identify the variant in *GCK*, then functional studies using a minigene approach allowed the functional characterization. **Results:** This study identified the synonymous variant, along with a nonsense mutation, c.859C>T (p.Gln287Ter), in *GCK* in two Italian patients. Minigene approach demonstrated that the synonymous variant disrupts splicing at the exon 5 boundary, leading to a frameshift and premature stop codon. Similarly, the nonsense mutation also altered splicing, exacerbating the molecular defect. **Conclusions:** These findings highlight the importance of functional assays, particularly minigene studies, in interpreting the pathogenicity of synonymous and nonsense variants, especially in genes like *GCK* where splicing alterations can significantly impact protein function. This study underscores the clinical utility of targeted genetic screening for personalized diabetes management.

## 1. Introduction

Monogenic diabetes is characterized by an impairment of glycemic control with an onset at an early age, typically before 25 years, although diagnosis may occur at older ages. The most common form of monogenic diabetes is maturity onset diabetes of the young (MODY, MIM #606391), accounting for 1–5% of subjects with hyperglycemia/diabetes mellitus in absence of β-cell autoantibodies [1,2].

Different from type 1 and type 2 diabetes, where multiple genetic causes and environmental factors are involved in the pathogenesis of the disease, MODYs are caused by mutations in genes involved in insulin production, and the condition is inherited with an autosomal dominant pattern. Currently, pathogenic variants in 14 different genes involved in glucose metabolism have been associated with the MODY phenotype. MODY2 (OMIM #125851) or *GCK*-MODY is caused by autosomal dominant mutations of the glucokinase gene (*GCK*, OMIM #138079). *GCK* encodes for an enzyme of 465 amino acids with a pivotal role in insulin secretion in response to blood glucose levels. *GCK*-MODY, *HNF1A*-MODY (OMIM #600496) and *HNF4A*-MODY (OMIM #125850) are the most common forms accounting for 95% of MODY cases [1,3]. Patients with *GCK*-MODY have mild, stable fasting hyperglycemia and the absence of treatment has no significant effect on their HbA1c or in the incidence of micro- and macrovascular complications compared to those who receive treatment. For this reason, except in specific circumstances (for instance, during pregnancy), a specific treatment is usually unnecessary in *GCK*-MODY patients, while in carriers of *HNF4a* and *HNF1a* defects, pharmacological treatment with oral hypoglycemic agents (generally sulphonylureas) is recommended [3,4].

Due to the widespread application of Next Generation Sequencing (NGS) in the routine molecular diagnostic of monogenic disorders, a growing number of *GCK* variants have been identified. To the best of our knowledge 947 different variants affecting *GCK* have been reported in the HGMD database (last accession 3 February 2025) and linked to a specific clinical phenotype. The interpretation of DNA variants and their classification according to the American College of Medical Genetics and Genomics (ACMG) guidance [5] represents a major challenge, especially when their biological impact is unknown. This is particularly relevant when the identified variants affect noncoding regions or are synonymous substitutions with strong genetic data supporting their causal role.

In this report, we provided the first functional validation of the known c.579G>T synonymous variant of the *GCK* gene [6,7] detected in a group of unrelated familial cases of *GCK*-MODY. By applying a minigene approach, this study moves beyond in silico predictions to offer direct experimental evidence of how this variant impacts *GCK* splicing.

## 2. Materials and Methods

**Molecular diagnosis**

Genomic DNA was obtained from probands and their relatives upon administration of informed consent. DNA was extracted from whole blood by using the Qiagen QIamp DNA Blood Midi (QIAGEN, Germantown, MD 20874, USA) kit according to the protocol provided by the manufacturer.

**Case 1 (BNT/515-23-D-0069):** Molecular screening was performed by analyzing an in silico panel of 45 genes involved in monogenic diabetes and dysglicemia [8] filtered from whole Exome Sequencing (ES) data. Around 50–100 ng of genomic DNA was used to sequencing target sequences using IDT xGen Exome Research Panel v2 enrichment kit (34 Mb, 19,433 genes) and Illumina technology (PE 2X150) on the Illumina platform Novaseq6000. Data were analyzed by a GATK-based toolkit. The presence of variants was confirmed by direct Sanger sequencing: PCR products were purified by enzymatic digestion with Exo/SAP-IT (Thermo Scientific, Waltham, MA, USA) and sequenced with the Big Dye Terminator Cycle Sequencing Kit (Thermo Scientific, MA, USA) according to the provided protocol; sequencing reactions were run on a 3130xl Genetic Analyzer (Thermo Scientific, MA, USA) and analyzed with the Sequencer 4.7 software (version 4.7; Gene Codes Corporation, Ann Arbor, MI, USA).

**Case 2 (SP23-D-0096):** Targeted molecular analysis of the *GCK* gene was performed by Sanger sequencing. Briefly, 100 ng of genomic DNA was used as a template to amplify by PCR the 10 coding exons of the *GCK* gene (OMIM #138079; RefSeq NM_000162.5; NP_000153.1) (oligonucleotides available upon request). PCR products were purified and sequenced as described above. Variants are reported according to the HGVS guidelines.

**In silico study analysis**

The sequences were analyzed by spliceAI [9] and the Human Splicing Finder (HSF, free license for academic users) [10], bioinformatic tools designed to predict eventual effects on splicing.

**Generation of the minigene plasmids**

The effect of the variants on the mRNA splicing process was evaluated by the minigene strategy, based on the pSPL3 exon trapping vector (already available in the Lab) [11,12]. A fragment of 458 bp (hg38: chr7: 44,189,283–44,189,740) comprising exon 5, intron 5, exon 6 and intronic flanking regions was amplified by PCR from genomic DNA of the proband 1; a sequence of 500 bp including exon 7 and intronic flanking regions (hg38: chr7: 44,187,097–44,187,596) was obtained from proband 2 and from a patient carrying the c.859C>A (p.Gln287Lys) used as negative control (primer sequences available upon request). The obtained PCR products were cloned in the PCR 2.1-TOPO TA (TOPO™ TA Cloning, Invitrogen, Carlsbad, CA, USA). Clones were checked by EcoRI restriction and by direct sequencing to verify and isolate both the WT and the mutated alleles. Finally, the fragments under analysis were subcloned into the splicing vector pSPL3 in the EcoRI restriction site and checked by sequencing.

**Cell culture, transient transfection and minigene sequences analysis**

The Hek-293 cell line was already available in the laboratory, previously purchased by ATCC. Cells were routinely cultured at 37 °C in a humidified atmosphere with 5% CO_2_, in the complete medium consisting of Essential Modified Medium (MEM) with 10% FBS. Transient transfections for the minigene assays were performed by seeding 8 × 10^5^/well Hek-293 cells in 6-well plates. The next day, the transfection mix composed of 2 µg of pSPL3 constructs and Lipofectamine 2000 reagent was added to cells, following the recommended protocol (Invitrogen, Thermo Fisher Scientific, MA, USA). Cells were collected after 24 h and processed for RNA extraction with the standard protocol of the RNeasy plus Mini Kit (Qiagen). Transfections were repeated twice with two different DNA preparations for each construct for 24 h and 48 h and all the RT-PCR products obtained verified by gel electrophoresis and Sanger sequencing.

The complementary DNA (cDNA) of the minigenes’ RNA was obtained by the retro-transcription of 1 µg of RNA by using Advantage^®^ RT-for-PCR (Takara, Kusastu, Shiga, Japan) and, subsequently, amplified by GoTaq Master mix (Promega, Madison, WI, USA) according to the manufacturer’s protocol (oligonucleotides sequences are available upon request). The PCR products were checked on 1.5% Agarose gel and cleaned up by Exo/SAP-IT (Thermo Scientific, Waltham, MA, USA) digestion. Sequences were obtained with the Big Dye Terminator Cycle Sequencing Kit according to the provided protocol and run on a 3130xl Genetic Analyzer (Thermo Scientific, Waltham, MA, USA). The analysis of the sequences was performed by the Sequencer 4.7 software (Gene Codes Corporation, Ann Arbor, MI, USA).

The sequences of all the oligonucleotide applied in this study are indicated in Table S2 (Supplementary Materials).

## 3. Results

### 3.1. Clinical Report

**Figure 1:**
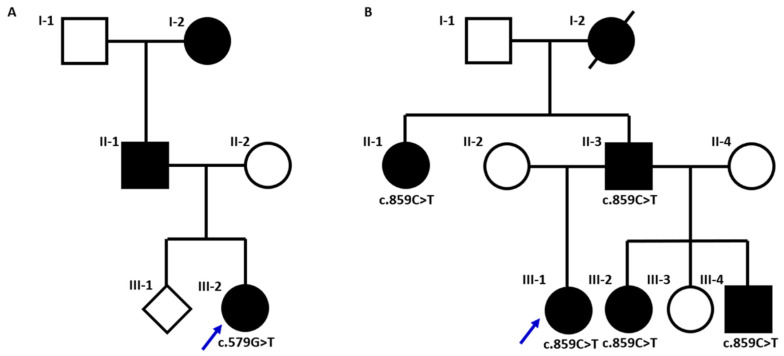
Pedigrees of the two familial cases with *GCK* variants. The results of the molecular testing indicate the presence of the c.579G>T p.(Gly193Gly) in case 1, panel (**A**), and of the c.859C>T p.(Gln287Ter) in case 2, panel (**B**). Genotypes are indicated below the tested members for each family. Black symbols indicate individuals with impaired fasting glucose; blue arrows indicate the two probands.

**Case 1 (BNT/515-23-D-0069).** A 20-year-old Caucasian woman was referred to our Endocrinology and Metabolism Unit due to decline of glycemic control, although hematochemical tests were not yet diagnostic for diabetes mellitus (DM), with a fasting plasma glucose of 121 mg/dL and a glycosylated hemoglobin (HbA1c) of 6.6% (Table 1). The patient reported mild fasting hyperglycemia since the age of 14, low birth weight (2.5 kg), normal pubertal development and a family history of DM on the paternal side (father and grandmother) (Figure 1). She denied chronic therapy intake. The patient was of normal weight and showed no clinical signs of hyperinsulinism, while lab tests demonstrated the absence of beta-cell autoimmunity. In light of strong suspicion of MODY, the patient underwent genetic analysis.

**Case 2 (SP23-D-0096).** A 57-year-old Caucasian woman came on the first visit at our Center due to DM, diagnosed 20 years before, in chronic therapy with metformin. The family history collection showed a brother with a diagnosis of MODY (Figure 1). She had a full-term pregnancy, during which she took insulin therapy for gestational diabetes mellitus; her daughter’s birth weight was about 3 kg. The patient had a normal weight. The latest hematochemical tests carried out under treatment with metformin, showed a fasting plasma glucose of 136 mg/dL, a HbA1c of 6.6% (Table 1) and a normal kidney function. Moreover, the glycemic diary reported fasting capillary blood glucose values between 130 and 140 mg/dL. There were no signs of diabetic retinopathy at the examination of fundus oculi nor signs of diabetic neuropathy during the physical examination. Therefore, as MODY diagnosis was likely, metformin therapy was discontinued and genetic testing was performed.

### 3.2. Molecular Characterization

Molecular diagnosis in Case 1 (BNT/515-23-D-0069) was performed by the analysis of an in silico panel of 45 genes involved in monogenic diabetes (Table S1 in Supplementary Materials) filtered from whole Exome Sequencing (ES) data. The analysis identified the synonymous substitution in exon 5 of the *GCK* gene, c.579C>T (p.Gly193Gly) (dbSNP, rs2128821570; ClinVar, VCV001683779.2), defined as a likely pathogenic according to ACMG criteria (PS4, PM2, PP3).

The synonymous variant, identified for the first time by our group, affects the last nucleotide of the last codon of the exon, and it has been previously found in the affected members of three unrelated families with impaired fasting glucose [6,7]. This variant was considered causative based on segregation data and an effect on the splicing of exon 5, through alteration of the adjacent donor site, was hypothesized. In silico analysis of this variant by dedicated bioinformatic tools such as spliceAI [9] and the Human Splicing Finder (HSF, free license for academic users) [10] indeed predicted an alteration through the loss of the canonical donor site with possible impairment of the exon 5 splicing (Table 2). We thus decided to perform a functional study by minigene approach.

The patient was also found to carry a heterozygous missense variant of *ABCC8* (OMIM#600509; RefSeq, NM_000352.6; NP_000343.2), the c.3100G>T (p.Ala1034Ser; dbSNP, rs1302864416) substitution in the 25 exon of the gene. This variant has never been reported in the literature and was interpreted as a variant of uncertain significance (VUS, PM2, PP2) according to ACMG criteria [5]. To date we could not verify the segregation of this variant.

Molecular diagnosis for Case 2 (SP23-D-0096) was performed by targeted Sanger sequencing of *GCK* and led to the identification of a nonsense substitution in the 7th coding exon of the gene, the c.859C>T (p.Gln287Ter) already reported in association with MODY/impaired fasting glucose [6,13]. This truncating variant introduces an early termination codon and is supposed to trigger a nonsense-mediated mRNA decay (NMD) of the carrying transcript. However, given the position of this codon, close to the exon/intron junction, we speculated about a possible effect on splicing. The effect of a severe variant introducing an early termination codon likely activating the NMD process or leading to the synthesis of a strongly altered, truncated protein, might be somehow mitigated by inducing a splicing defect. Indeed, the transcription of an altered mRNA carrying an in-frame exon skipping or arising from the activation of cryptic splicing sites leading to the in-frame loss or inclusion of nucleotides may allow the synthesis of a partially functional protein.

Evaluation of the c.859C>T (p.Gln287Ter) nonsense substitution by bioinformatic tools predicted a possible alteration of the exon 7 splicing by affecting an auxiliary, regulatory exonic sequence (Table 2). We thus decided to include this variant in the functional assay.

### 3.3. Functional Studies

**Figure 2:**
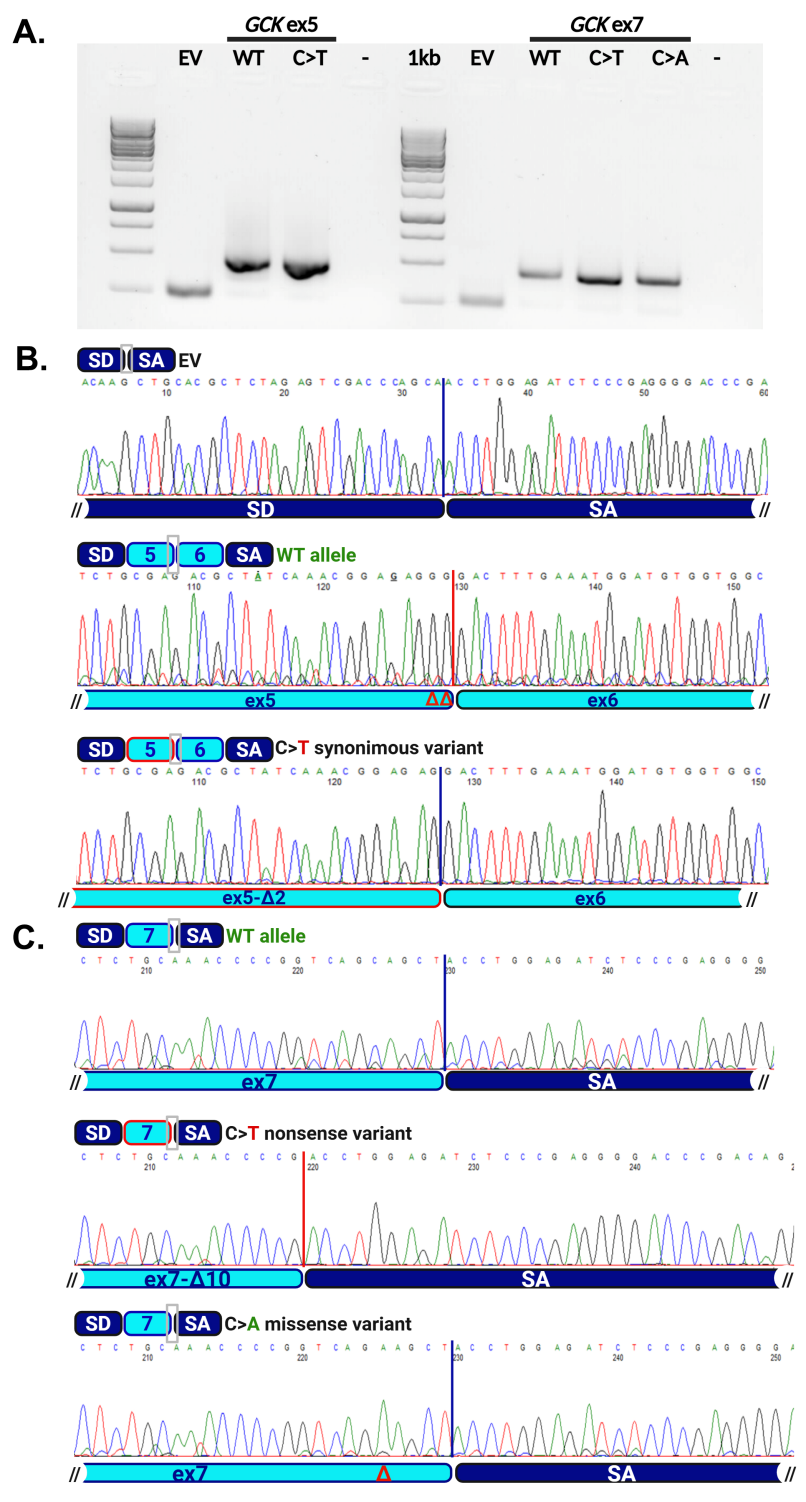
Minigene approach for the identified *GCK* variants. (**A**) RT-PCR product obtained from cells transfected with the different splicing constructs. Specific bands of 263 bp, 459 bp and 500 bp corresponding to an expected WT splicing were detected in cells transfected with the empty pSPL3 vector (EV), with exon 5 and 7 wild-type constructs, respectively (GCKex 5 and GCKex7 WT). Bands obtained in cells transfected with the different mutated constructs were only apparently of the same size as the WT, as the aberrant splicing caused by their presence demonstrated a difference of few nucleotides not appreciable by standard agarose electrophoresis. (**B**,**C**) Chromatograms showing the sequence of the observed splicing products at the boundaries between exons, as indicated by the schematic representations above sequences. 1kb, molecular weight marker; EV, empty vector; WT, wild-type allele; -, PCR negative control; SD and SA, pSPL3 exons; Δ symbols indicate the nucleotides lost due to the splicing defects. Figure composed by Biorender.com.

To confirm the predicted effects on the splicing of the identified variants, we designed a minigene approach. This strategy is based on the use of the pSPL3 splice vector [11,12], an expression plasmid generated for exon trapping and suitable to verify the effects of DNA variants on splicing. We thus generated different expression constructs: for the c.579G>T (p.Gly193Gly) variant, we subcloned into the pSPL3 vector a PCR product containing a genomic region, both wild-type (WT) and mutated, spanning *GCK* exons 5 and 6, and comprising the whole intervening sequence between them and the flanking intronic sequences upstream exon 5 and downstream exon 6. For exon 7, we subcloned a genomic region spanning the exon and the intronic flanking sequences. As the impact of substitutions on the function of regulatory elements (e.g., Exonic Splicing Enhancer or Silencer, ESE and ESS) which are auxiliary for splicing are strongly sequence-dependent, we generated a control construct carrying the c.859C>A (p.Gln287Lys) substitution, previously identified in a patient with impaired fasting glucose [6], and affecting the same codon in a different position, predicted not to alter the splicing of exon 7 (Table 2). The different constructs were transiently transfected in Hek-293 cells and total RNA was extracted after 24 h; RT-PCR with oligonucleotides specific for the vector exons was then performed. The products obtained from cells transfected with the empty vector (EV) and with the different WT and mutated constructs are shown in Figure 2A. For EV and WT plasmids, we detected bands with the size expected from a normal splicing event combining the artificial exons of the vector in EV-transfected cells and generating a minigene with vector and *GCK* exons in WT-transfected cells. A PCR product with a similar size to that observed for WT was obtained from cells transfected with mutated constructs. However, the Sanger sequencing demonstrated that, in accordance with the in silico predictions, both the c.579G>T (p.Gly194Gly) and c.859C>T (p.Gln287Ter) substitutions altered the splicing. The first one led to the generation of a novel donor site leading to the loss of the two last nucleotides of exon 5 (Figure 2B); the c.859C>T caused the formation of a novel donor site masking the canonical one with loss of the last 10 nucleotides of exon 7 (Figure 2C). Both variants thus caused a frameshift with the formation of an early termination codon. The c.859C>A (p.Gln287Lys) variant of the exon 7, used as a control, did not alter the splicing as predicted by the bioinformatic tools.

## 4. Discussion

Monogenic diabetes, a form of diabetes caused by mutations in a single gene, is a distinct subset of diabetes which is frequently characterized by onset of hyperglycemia at an early age, although diagnosis may occur at older ages. Among various types of monogenic diabetes, MODY is the most common with *GCK*-MODY affecting around 30% of cases [1]. As indicated, it is caused by alteration of *GCK* , a gene playing a pivotal role in insulin secretion in response to blood glucose levels [1,2,7].

Based on the data published on the free public HGMD database (https://www.hgmd.cf.ac.uk/ac/gene.php?gene=GCK, last accessed on 3 February 2025), missense variants account for 71% of pathogenic mutation and represent the most common cause of *GCK*-MODY. In recent years, several synonymous *GCK* were reported in the literature and linked to the clinical phenotype [14], but to the best of our knowledge, the functional study of the c.579G>T (p.Gly193Gly) has never been reported.

Synonymous variants were considered benign for a long time. In recent years, on the contrary, their pathogenic role has been increasingly recognized. A substitution in a coding sequence which does not result in aminoacidic change may exert its role by different mechanisms including alteration of splicing and impairment of the transcription and translation efficiency (e.g., impact on regulatory sequences; variable abundance of different aminoacyl tRNAs; generation or modification of secondary structures) (Oelschlaeger, 2024 PMID: 38275761, [15]), thus impacting the expression of the gene. By applying a minigene approach, we demonstrated that the c.579G>T variant disrupts splicing at the boundary of exon 5, resulting in the creation of a novel donor splice site, as predicted by the in silico analysis. This leads to the loss of the last two nucleotides of exon 5 with alteration of the frame in the cDNA sequence, generation of an early Stop codon and possible targeting of the mutated transcript to nonsense-mediated decay (NMD). The fraction of transcript eventually escaping to NMD would lead to the synthesis of a truncated GCK protein. This variant was identified for the first time by our group in three unrelated patients with impaired fasting glucose [6] for a total of five unrelated familial cases, all coming from the same geographical area in Liguria, a small region in the north of Italy. This may suggest the existence of some peculiar, population-specific genetic features as in the same region we identified six unrelated patients with other recurrent *GCK* variants (see the recurrent “pesto mutation” in/del [16]).

The BNT/515-23-D-0069 patient was also found to carry a heterozygous VUS in the *ABCC8* gene (OMIM #600509) c.3100G>T (p.Ala1034Ser). The gene is involved in different dysglicemic conditions including diabetes mellitus and hyperinsulinemic hypoglycemia, with or without neurological and neurodevelopmental signs, and with different patterns of inheritance (AR or AD).

Digenic inheritance of MODY pathogenic variants is probably more common than reported in the literature [17]. A combination of a variant in the *GCK* and a heterozygous VUS in *ABCC8* has been recently reported in a case of congenital hyperinsulinism during infancy. Whereas the *GCK* variant was regarded as the underlying cause for disease in this proband, the authors speculated about a possible role of the *ABCC8* variant in modulating the severity of the phenotype [18]. In the case reported here, in the absence of further genetic (segregation) or functional data, and no occurrence of peculiar clinical features, we could not draw any conclusion about the possible contribution, if any, of this variant.

The two patients described in this manuscript carrying the synonymous variant presented with clinical features consistent with *GCK*-MODY, including impaired fasting glucose (IFG) and a family history of diabetes [1,2]. Both patients had only mild increase in HbA1c, which is another feature of *GCK*-MODY, with a glycated hemoglobin usually within range of 5.6–7.6% [19]. Both patients had no signs of β-cell autoimmunity, distinguishing their condition from type 1 diabetes, and they did not require intensive insulin therapy, which is often a feature of type 1 and type 2 diabetes. This agrees with the milder nature of *GCK*-MODY, where hyperglycemia is usually present, but the condition can be managed mostly without pharmacological intervention [3]. The two patients, moreover, had no signs of complications related to diabetes, such as retinopathy or neuropathy, as *GCK*-MODY is rarely associated with micro- and macrovascular complications of diabetes. In the second case (SP23-D-0096), we identified the c.859C>T (p.Gln287Ter) nonsense mutation of *GCK*, which is a known pathogenic variant in association with MODY2 [6,7,13]. This variant introduces a premature stop codon in a region of the *GCK* cDNA which is predicted to trigger NMD of the resulting mRNA transcript, preventing the synthesis of an aberrant/truncated protein. In this context, there is growing evidence that besides the generation of a PTC, in specific cases, a nonsense-associated altered splicing might occur and upregulate transcripts that have skipped the sequences carrying the newly formed stop codon with maintenance of the cDNA reading frame and the synthesis of partially functional protein [20]. Although to the best of our knowledge this has not yet been described for *GCK*, this phenomenon has been associated with both physiological and pathological conditions [20,21,22,23]. This event may add complexity to the functional alteration caused by the variant and somehow mitigate the negative effect of the generated Stop codon by inducing a favorable, yet altered splicing, with maintenance of the cDNA reading frame and partially functional synthesis [20]. Unfortunately, this is not the case we are reporting, as the splicing alteration indeed induced by the nonsense c.859C>T (p.Gln287Ter) variant reinforces the molecular defect, as the consequent skipping of the *GCK* exon 7 causes a frameshift and generation of an early termination codon.

## 5. Conclusions

In conclusion, our study demonstrated that the p.Gly193Gly synonymous variant in *GCK* compromises splicing and contributes to the development of *GCK*-MODY. It is important to note, however, that these findings are specific to this particular variant; they do not support a broad generalization to other *GCK* mutations. Because the functional impact of missense, synonymous, and noncanonical splice site variants can vary significantly, each VUS must be individually evaluated. Despite improvements in AI-driven bioinformatic predictions, trained on an increasing amount of data from experimentally validated cases, functional studies remain the only definitive way to confirm pathogenicity. In this context, the minigene approach may be essential for assessing the impact of genetic variants that do not alter the protein sequence directly but may still disrupt gene function through effects on mRNA splicing, particularly when primary cells expressing the gene of interest are not available from patients for transcript analyses.

While minigene assays are robust tools for studying splice-site selection, they may not fully recapitulate the complex endogenous regulation of *GCK* splicing in vivo. Unlike the native genomic locus, minigenes typically lack full-length introns, distal enhancer elements, and the native chromatin context, all of which can influence the recruitment of splicing factors. Moreover, as splicing may be affected by the specific cellular context, such as the use of neutral cellular recipients for the minigene assay, such as Hek-293, while the minigene assay can efficiently identify the potential for a variant to disrupt splicing, the exact ratio of spliced isoforms observed may differ from those in patient-specific tissues, such as pancreatic beta cells or hepatocytes. However, this method has been already successfully applied to assess the role of rare intronic or synonymous variants of the *GCK* gene in MODY patients [14]. Moreover, it is accepted beside the analysis of RNA and/or complementary DNA derivatives from patient’s primary cells as a functional test allowing the variant interpretation and scoring according to the ACMG and the specific guidelines for the *GCK* gene [5,24]. By identifying individuals who carry specific *GCK* mutations, clinicians could diagnose *GCK*-MODY earlier, enabling timely intervention and more personalized management strategies. Obtaining a genetic diagnosis, indeed, leads to discontinuation of unnecessary treatment for patients with *GCK*-MODY, as well as allowing them to switch from injectable insulin treatment to convenient oral treatment in *HNF1A*-MODY and *HNF4A*-MODY. Also, it guides clinicians to adjust the timeline and frequency of follow up for diabetes complications because of the significantly different risk of sequelae across different MODY forms [25].

## Figures and Tables

**Table 1:** Synopsis of the clinical features of the reported patients.

Patient	Sex	Age	Birth Weight	Age at Diagnosis	FPG	HbA1c	GDM	Other Affected Family Member	Treatment
Case 1	F	22 ys	2500 g	15 ys	IFG	6.6%	No	Yes	No
Case 2	F	60 ys	ND	37 ys	DM	6.6%	Yes	Yes	Yes

FPG, fasting plasma glucose: impaired fasting glucose [IFG] = 100–125 mg/dL; diabetes mellitus [DM] ≥ 126 mg/dL; HbA1c glycosylated hemoglobin (reference value: 4.3–5.9%); GDM, gestational diabetes mellitus; ND, not determined.

**Table 2:** In silico analysis of *GCK* variants.

*GCK* Variant IDs (dbSNP, ClinVar)	RefSeq NM_000162.5 NP_000153.1		spliceAI (Δ Score)	HSF
rs2128821570 VCV001683779.2	c.579G>T	p.(Gly193Gly)	0.50 Donor loss; 0.99 donor gain	Alteration of the WT Donor site, most probably affecting splicing
-	c.859C>T	p.(Gln287Ter)	0.62 Donor loss	Alteration of an auxiliary sequence
-	c.859C>A	p.(Gln287Lys)	0.19 Donor loss	None

spliceAI, Delta (Δ) score ranges from 0 to 1 and represents the probability that a variant in a given position can affect splicing in a window 500 bp (default) around it. Three different cut-offs are applied: 0.2 (high recall), 0.5 (recommended), and 0.8 (high precision) [9]. HSF, Human Splicing Finder (Genomnis SAS, Human Splicing Finder Web Site, version 2.37; https://www.genomnis.com/) [10].

## Data Availability

The data that support our findings in this study are available from the corresponding author upon reasonable request.

## Supplementary Materials

The following supporting information can be downloaded at: https://www.mdpi.com/article/10.3390/genes17020214/s1, Table S1: List of the genes included in the in silico panel filtered from whole ES data; Table S2: Name and sequences of the oligonucleotides used in this study.

## Abbreviations

The following abbreviations are used in this manuscript:

MODY	Maturity onset diabetes of the young
GCK-MODY	MODY type 2
GCK	Glucokinase gene
NGS	Next Generation Sequencing
ACMG	American College of Medical Genetics and Genomics
ES	Exome Sequencing
cDNA	Complementary DNA
DM	Diabetes mellitus
HbA1c	Glycosylated hemoglobin
NMD	Nonsense-mediated mRNA decay
WT	Wild-type
EV	Empty vector

## References

[1] Nkonge K.M., Nkonge D.K., Nkonge T.N. (2020). The Epidemiology, Molecular Pathogenesis, Diagnosis, and Treatment of Maturity-Onset Diabetes of the Young (MODY). Clin. Diabetes Endocrinol..

[2] Younis H., Ha S.E., Jorgensen B.G., Verma A., Ro S. (2022). Maturity-Onset Diabetes of the Young: Mutations, Physiological Consequences, and Treatment Options. J. Pers. Med..

[3] Delvecchio M., Pastore C., Giordano P. (2020). Treatment Options for MODY Patients: A Systematic Review of Literature. Diabetes Ther..

[4] Broome D.T., Pantalone K.M., Kashyap S.R., Philipson L.H. (2021). Approach to the Patient with MODY-Monogenic Diabetes. J. Clin. Endocrinol. Metab..

[5] Richards S., Aziz N., Bale S., Bick D., Das S., Gastier-Foster J., Grody W.W., Hegde M., Lyon E., Spector E. (2015). Standards and Guidelines for the Interpretation of Sequence Variants: A Joint Consensus Recommendation of the American College of Medical Genetics and Genomics and the Association for Molecular Pathology. Genet. Med..

[6] Aloi C., Salina A., Minuto N., Tallone R., Lugani F., Mascagni A., Mazza O., Cassanello M., Maghnie M., d’Annunzio G. (2017). Glucokinase Mutations in Pediatric Patients with Impaired Fasting Glucose. Acta Diabetol..

[7] Delvecchio M., Mozzillo E., Salzano G., Iafusco D., Frontino G., Patera P.I., Rabbone I., Cherubini V., Grasso V., Tinto N. (2017). Monogenic Diabetes Accounts for 6.3% of Cases Referred to 15 Italian Pediatric Diabetes Centers During 2007 to 2012. J. Clin. Endocrinol. Metab..

[8] Aloi C., Salina A., Caroli F., Bocciardi R., Tappino B., Bassi M., Minuto N., d’Annunzio G., Maghnie M. (2023). Next Generation Sequencing (NGS) Target Approach for Undiagnosed Dysglycaemia. Life.

[9] Jaganathan K., Kyriazopoulou Panagiotopoulou S., McRae J.F., Darbandi S.F., Knowles D., Li Y.I., Kosmicki J.A., Arbelaez J., Cui W., Schwartz G.B. (2019). Predicting Splicing from Primary Sequence with Deep Learning. Cell.

[10] Desmet F.-O., Hamroun D., Lalande M., Collod-Béroud G., Claustres M., Béroud C. (2009). Human Splicing Finder: An Online Bioinformatics Tool to Predict Splicing Signals. Nucleic Acids Res..

[11] Duyk G.M., Kim S.W., Myers R.M., Cox D.R. (1990). Exon Trapping: A Genetic Screen to Identify Candidate Transcribed Sequences in Cloned Mammalian Genomic DNA. Proc. Natl. Acad. Sci. USA.

[12] Burn T.C., Connors T.D., Klinger K.W., Landes G.M. (1995). Increased Exon-Trapping Efficiency through Modifications to the pSPL3 Splicing Vector. Gene.

[13] Thomson K.L., Gloyn A.L., Colclough K., Batten M., Allen L.I.S., Beards F., Hattersley A.T., Ellard S. (2003). Identification of 21 Novel Glucokinase (GCK) Mutations in UK and European Caucasians with Maturity-Onset Diabetes of the Young (MODY). Hum. Mutat..

[14] Tiulpakov A., Zubkova N., Makretskaya N., Krasnova T.S., Melnikova A.I., Fedyaeva A.S., Vasilyev E., Petrov V.M., Rubtsov P.M. (2020). Minigene Splicing Assessment of 20 Novel Synonymous and Intronic Glucokinase Gene Variants Identified in Patients with Maturity-onset Diabetes of the Young. Hum. Mutat..

[15] Oelschlaeger P. (2024). Molecular Mechanisms and the Significance of Synonymous Mutations. Biomolecules.

[16] Salina A., Bassi M., Aloi C., Strati M.F., Bocciardi R., d’Annunzio G., Maghnie M., Minuto N. (2023). “Pesto” Mutation: Phenotypic and Genotypic Characteristics of Eight GCK/MODY Ligurian Patients. Int. J. Mol. Sci..

[17] Patouni K., Cinek O., Pruhova S., Elblova L., Xatzipsalti M., Sertedaki A., Vazeou A. (2021). A Case of Digenic Maturity Onset Diabetes of the Young with Heterozygous Variants in Both HNF1A and HNF1Β Genes. Eur. J. Med. Genet..

[18] Velde C.D., Molnes J., Berland S., Njølstad P.R., Molven A. (2025). Clinical and Genetic Characteristics of Congenital Hyperinsulinism in Norway: A Nationwide Cohort Study. J. Clin. Endocrinol. Metab..

[19] Steele A.M., Wensley K.J., Ellard S., Murphy R., Shepherd M., Colclough K., Hattersley A.T., Shields B.M. (2013). Use of HbA1c in the Identification of Patients with Hyperglycaemia Caused by a Glucokinase Mutation: Observational Case Control Studies. PLoS ONE.

[20] Haque B., Cheerie D., Birkadze S., Xu A.L., Nalpathamkalam T., Thiruvahindrapuram B., Walker S., Costain G. (2024). Estimating the Proportion of Nonsense Variants Undergoing the Newly Described Phenomenon of Manufactured Splice Rescue. Eur. J. Hum. Genet..

[21] Lambert J.M., Ashi M.O., Srour N., Delpy L., Saulière J. (2020). Mechanisms and Regulation of Nonsense-Mediated mRNA Decay and Nonsense-Associated Altered Splicing in Lymphocytes. Int. J. Mol. Sci..

[22] Abrahams L., Savisaar R., Mordstein C., Young B., Kudla G., Hurst L.D. (2021). Evidence in disease and non-disease contexts that nonsense mutations cause altered splicing via motif disruption. Nucleic Acids Res..

[23] Aznarez I., Zielenski J., Rommens J.M., Blencowe B.J., Tsui L.C. (2007). Exon skipping through the creation of a putative exonic splicing silencer as a consequence of the cystic fibrosis mutation R553X. J. Med. Genet..

[24] ClinGen Monogenic Diabetes Expert Panel Specifications to the ACMG/AMP Variant Interpretation Guidelines for GCK Version 3.1.0. https://cspec.genome.network/cspec/ui/svi/doc/GN086#pmid_30096381.

[25] Tosur M., Philipson L.H. (2022). Precision Diabetes: Lessons Learned from maturity-onset Diabetes of the Young (MODY). J. Diabetes Investig..

