# Supplementary material for: Deciphering Silence: Functional Studies of GCK Synonymous and Nonsense Variants and Their Importance in Understanding Diabetes
Source: Genes (Basel). 2026 Feb 10;17(2):214. doi: 10.3390/genes17020214 (PMC12941200; doi:10.3390/genes17020214)
Supplement: Supplementary file 1 [file genes-17-00214-s001.zip › genes-4055023-supplementary.pdf]

Table S1. List of the genes included in the *in silico* panel filtered from whole ES data.

N.	OMIM ID	Gene symbol
1	600509	*ABCC8*
2	607358	*AIRE*
3	606844	*ALMS1*
4	604299	*APPL1*
5	107777	*AQP2*
6	300538	*AVPR2*
7	209901	*BBS1*
8	191305	*BLK*
9	611507	*CISD2*
10	602121	*DIAPH1*
11	600576	*GATA4*
12	601656	*GATA6*
13	138079	*GCK*
14	121011	*GJB2*
15	610192	*GLIS3*
16	138130	*GLUD1*
17	601609	*HADH*
18	142410	*HNF1A*
19	189907	*HNF1B*
20	600281	*HNF4A*
21	147730	*IL2RA*
22	176730	*INS*
23	147670	*INSR*
24	600937	*KCNJ11*
25	603301	*KLF11*
26	606453	*LRBA*
27	605283	*MAGEL2*
28	601724	*NEUROD1*
29	605290	*OPA1*
30	606580	*OPA3*
31	167413	*PAX4*
32	607108	*PAX6*
33	600733	*PDX1/IPF1*
34	300039	*POU3F4*
35	612659	*RFX6*
36	602329	*SEL1L*
37	608937	*SH2B1*
38	182381	*SLC5A2*
39	608160	*SOX9*
40	610928	*SOX17*
41	600555	*STAT1*
42	102582	*STAT3*
43	604260	*STAT5B*
44	612988	*TMEM126*
45	606201	*WFS1*

Note: This panel includes genes associated with different forms of dysglicemia, according to the literature and websites related to genomic tests (Genomics England PanelApp; OMIM), and including monogenic diabetes; Familial diabetes; Hereditary isolated diabetes insipidus; neonatal diabetes; Diabetes with additional phenotypes suggestive of a monogenic aetiology.

Table S2. Name and sequences of the oligonucleotides used in this study.

Oligonucleotides applied to validate *GCK* variants and to prepare minigenes	
**Name**	**Sequence**
GCK-ex5-6F	TGCAGGAGGTAGTGACAGGC
GCK-ex5-6R	CACAGGGCCCTTGAAGC
GCK_5_F	CAAGGAGAATCGTTCCCAAA
GCK_5_R	GCCGCCAAGGAGAAAGGCAG
GCK-ex7F	AGCAACCCAGGTCTTCCAG
GCK-ex7R	CAGAAGGGATGGAGCTTACG
**Oligonucleotides used to amplify cDNA in minigene assay**	
**Name**	**Sequence**
SA2	ATCTCAGTGGTATTTGTGAGC
SD6	TCTGAGTCACCTGGACAACC
